# Nanostructure-free crescent-shaped microparticles as full-color reflective pigments

**DOI:** 10.1038/s41467-023-36482-4

**Published:** 2023-02-11

**Authors:** Yi Yang, Jong Bin Kim, Seong Kyeong Nam, Mengmeng Zhang, Jiangping Xu, Jintao Zhu, Shin-Hyun Kim

**Affiliations:** 1 Department of Chemical and Biomolecular Engineering, Korea Advanced Institute of Science and Technology (KAIST), Daejeon, 34141 Korea; 2 Key Laboratory of Material Chemistry for Energy Conversion and Storage, Ministry of Education, School of Chemistry and Chemical Engineering, Huazhong University of Science and Technology (HUST), Wuhan, 430074 China

**Keywords:** Materials for optics, Polymers, Optical materials and structures

## Abstract

Structural colors provide a promising visualization with high color saturation, iridescent characteristics, and fade resistance. However, pragmatic uses are frequently impeded by complex manufacturing processes for sophisticated nanostructures. Here, we report a facile emulsion-templating strategy to produce crescent-shaped microparticles as structural color pigments. The micro-crescents exhibit brilliant colors under directional light originating from total internal reflections and optical interferences in the absence of periodic nanostructures while being transparent under ambient light. The colors are finely tunable by adjusting the size of the micro-crescents, which can be further mixed to enrich the variety. Importantly, the pre-defined convex surface secures high stability of colors and enables structural coloration on target surfaces through direct deposition as inks. We anticipate this class of nanostructure-free structural colorants is pragmatic as invisible inks in particular for anti-counterfeiting patches and color cosmetics with distinctive impressions due to low-cost, scalable manufacturing, unique optical properties, and versatility.

## Abstract (web summary)

Structural colors arise from nanostructures or special microgeometries. Here, the authors report on crescent-shaped microparticles as full-color reflective pigments that show brilliant colors in directional light and are transparent in ambient light.

## Introduction

Structural coloration is an intriguing optical phenomenon arising from wavelength-selective interference, diffraction, or scattering of light through light-matter interaction^1–3^. Nature has created numerous structures for structural coloration during long-term evolution for reproduction, camouflage, and signal transmission^4–8^. In contrast to the pigmentary colors, structural colors show preponderances of fade resistance, eco-friend, high saturation, iridescence, and responsiveness^9–12^. To utilize these unique properties, periodic sub-wavelength structures with photonic bandgaps have been artificially produced for structural coloration^13–16^, which have been extensively explored for various applications in sensors, displays, security, and information storage^17–19^. In particular, the periodic structures are tailored to have a granular format with appropriate confinements to produce structural-color pigments^20–22^. However, the production of periodic structures at sub-wavelength scales suffers from delicate protocols and limited throughput for both top-down and bottom-up approaches^23–26^.

Recently, it has been thoughtfully demonstrated that iridescent structural colors can be developed by curved interfaces of biphasic droplets without any periodic nanostructures through the synergic effect of total internal reflection (TIR) and optical interference^27^. In addition, gold nanoparticles, assembled around the rims of the curved interfaces, funnel the lights by scattering, providing enhanced and isotropized structural colors^28^. Following the pioneering works, retroreflective structural-color films and micropatterns have been designed by partially embedding microspheres in a transparent tape or directly printing micro-domes through the ink-jet technique^29–34^. Although high visibility and high resolution have been achieved, the colors are restrained on planar and transparent panels as the curved structures are required on the opposite side. More flexible and direct utilization of such structural color still remains an important challenge. To address the current challenge, it is preferred to produce well-defined microparticles with curved surfaces in advance for the formulation of inks in a form of suspension. Although biphasic droplets show structural colors, their low stability and high production cost prevent their use as ink additives. Therefore, it is highly demanding to produce stable solid microparticles responsible for structural coloration through TIR and interference through a simple and scalable process.

Herein, we report a facile means to create iridescent pigments of crescent-shaped microparticles with a well-defined curved interface, referred to as micro-crescents. Despite the extensive works on the microfluidic production of hemispherical or crescent microparticles, iridescent reflection has never been identified due to the relatively large size of approximately 100 μm^35–38^. To produce the micro-crescents with a radius responsible for TIR and interference through a scalable process, we employ evaporation-induced phase separation of polymer and silicone oil from a homogenous mixture in single-emulsion droplets. The polymer and silicone oil are dissolved in a co-solvent of chloroform, which is microfluidically emulsified in water. As the co-solvent is depleted by evaporation, polymer and silicone oil are phase-separated and evolve into a paired droplet with minimum interfacial energy. Removal of the silicone oil from the consolidated droplets leaves behind the polymeric micro-crescents with strongly convex and weakly concave surfaces. As the micro-crescents are produced by evaporation-induced phase separation and consolidation from single-phase droplets, the production is scalable and a small dimension is easy to achieve. The micro-crescents show TIR along the convex surface and display structural colors through interference which are readily tunable by adjusting the radius of micro-crescents in the range of 5–20 μm. The colors can be further diversified by mixing the micro-crescents with different sizes, in a similar manner to chemical pigments in a palette. Distinctively, the micro-crescents selectively disclose vivid colors under directional lights while being transparent under ambient lights, which is particularly appealing as invisible inks for anti-counterfeiting applications. Moreover, the orientation of micro-crescents can be unified by utilizing strong adhesion of weakly concave surfaces to flat substrates relatively to strongly convex ones, providing enhanced color saturation and Janus characteristics of color panels.

## Results

### Production of micro-crescents through phase separation in emulsion droplets

**Fig. 1:**
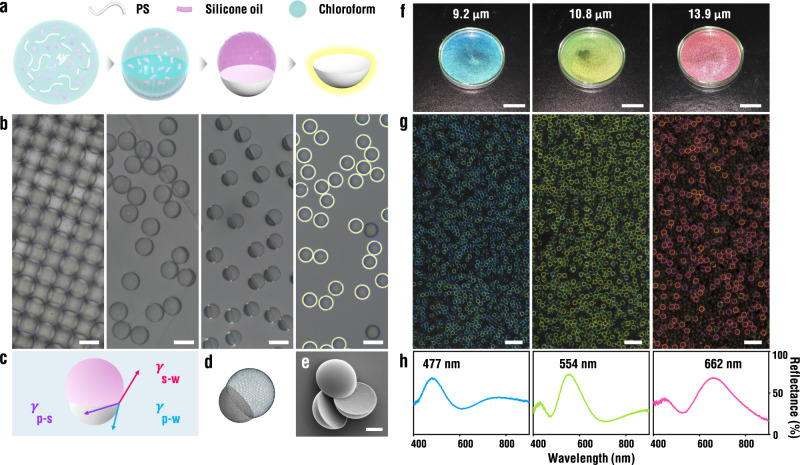
Micro-crescents produced by phase separation in emulsion droplets. **a, b** Sets of cartoon and reflection optical micrographs showing the phase separation by evaporation of chloroform and production of micro-crescents through the removal of silicone oil. Scale bar, 50 μm. **c** Cartoon of a paired drop, where interfacial tensions are balanced as represented with arrows. **d** Model structure of the paired drop at minimum interfacial energy state calculated using *Surface Evolver*. **e** Scanning electron microscope (SEM) image of the micro-crescents. Scale bar, 10 μm. **f** Sets of photographs for the aqueous suspensions of micro-crescents with an average radius of 9.2, 10.8, and 13.9 μm, respectively. Scale bar, 20 mm. **g, h** Reflection optical micrographs and reflectance spectra corresponding to (**f**). Scale bar, 100 μm. Source data for **h** is provided as a Source Data file.

Micro-crescents are produced by three main steps: (1) preparation of monodisperse emulsion droplets, (2) phase separation between polymer and silicone oil, and (3) selective removal of silicone oil. A mixture of polystyrene (PS) and silicone oil in a volume ratio of 2:7 dissolved in a co-solvent of chloroform is emulsified in an aqueous solution of poly(vinyl alcohol) (PVA) using a capillary microfluidic device to have a uniform diameter and composition, where the diameter is controllable by adjusting flow rates (Supplementary Fig. 1). The evaporation of the co-solvent leads to spontaneous separation into the PS-rich phase and the silicone oil-rich phase to form biphasic droplets due to the low miscibility while reducing the size of droplets^39,40^. The PS is completely consolidated as the chloroform is depleted from the droplets whereas the silicone oil remains as a liquid. Therefore, the silicone oil can be removed from the PS micro-crescents by mechanical agitation through ultrasonication (Fig. 1a, b). With the interfacial energies among PS, silicone oil, and water with PVA experimentally measured, the final morphology of the paired droplets is reproduced using *Surface Evolver*^41^, indicating that the shape is predominantly dictated by the minimum surface energy state (Fig. 1c, d and Supplementary Fig. 2)^42,43^.

The micro-crescents are composed of a strongly convex surface and a weakly concave surface (Fig. 1e and Supplementary Fig. 3). The angle of the spherical convex surface relative to the basal plane is approximately measured as 81° and the angle of the concave surface is 20° from the observation (Supplementary Fig. 4). The micro-crescents on the substrate predominantly have two different orientations of either concave-surface-up or convex-surface-up, under the action of gravity, due to the morphology. When illuminated under white light for microscopic observation, each micro-crescent exhibits a pronounced color ring along the annular edge from the concave side, while showing no reflection from the convex side (Fig. 1b). The colors of the rings vary along with the radius of the micro-crescents. For example, the micro-crescents with an average radius of 9.2, 10.8, and 13.9 μm display blue, green, and red colors, respectively, which are also consistent with the macroscopic observation of the suspensions with naked eyes under directional illumination (Fig. 1f, g). The reflectance spectra show a primary peak at the wavelength responsible for the structural color, respectively (Fig. 1h). High size uniformity of the micro-crescents guaranteed by microfluidics minimizes the color variation (Supplementary Fig. 5).

### Colors from micro-crescents

**Fig. 2:**
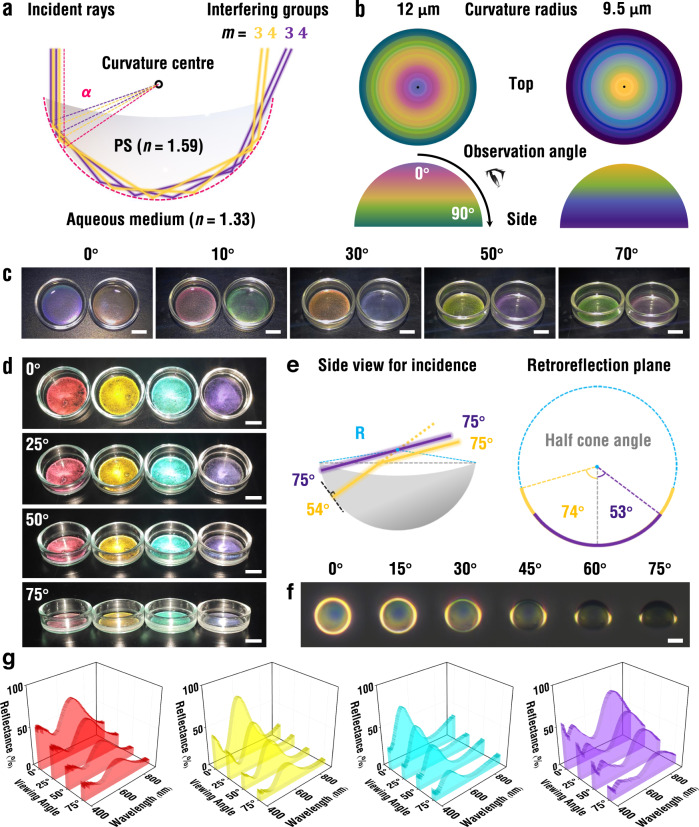
Structural colors from micro-crescents. **a** Illustration showing two different pairs of light paths responsible for total internal reflection (TIR) and optical interference in the micro-crescent. **b** Top-view and side-view color maps for the curved interfaces with curvature radii (CRs) of 12 μm and 9.5 μm, where the colors are converted from the reflectance spectra calculated by a model previously reported. **c** Series of photographs showing the color change along with the observation angle from 0° to 70° under incident light normal to the suspensions of micro-crescents with CRs of 15 μm (left) and 12.5 μm (right). Scale bar, 10 mm. **d** Series of photographs showing the invariant colors with the observation angle from 0° to 75° of the suspensions of micro-crescents with a radius of 13.1, 11.6, 9.5, 8.0 μm at retroreflection condition. Scale bar, 10 mm. **e** Cartoons for the trajectory of light path and plane responsible for retroreflection without (purple) and with (yellow) refraction of light at the concave surface. The refraction increases the half cone angle on the retroreflection plane from 53° to 74° for the incident angle of 75°. **f** Reflection optical micrographs of a micro-crescent with a radius of 11.6 μm at various tilting angles. Scale bar, 10 μm. **g** Reflectance spectra corresponding to (**d**). Source data for **g** is provided as a Source Data file.

The annular structural color developed in each micro-crescent is caused by optical interference among rays reflected at the convex surface by TIR (Fig. 2a). For the incident beam perpendicular to the basal plane from the concave side, rays that travel near the periphery are selectively guided by multiple TIR owing to the refractive index (RI) transition from PS (*n* = 1.59) to water (*n* = 1.33); the critical angle for the TIR is *α* = 56.8° (Supplementary Fig. 6)^44^. That is, the rays impinging into the micro-crescent with an incident angle greater than *α* are reflected by multiple times without a loss, of which pairs with different numbers of reflection (*m*) yet the same outgoing direction from the micro-crescent interfere to develop the reflection colors. Therefore, the colors are developed in a form of a ring along the periphery of each micro-crescent when observed from the concave side.

The pairs of the rays responsible for the optical interference are selected by the outgoing direction from the micro-crescent (see pairs of yellow and violet trajectories in Fig. 2a, for example). Therefore, the structural colors vary with the observation angle for fixed incident light normal to the suspensions (Fig. 2b, c). For example, the suspension of micro-crescents with an average radius of 14.8 μm (curvature radius (CR) = 15 μm) shows purple at retroreflection with an observation angle of 0°, which gradually turns to red, orange, yellow, and green as the polar angle increases to 70° (the left sample of Fig. 2c). The micro-crescents with an average radius of 12.3 μm (CR = 12.5 μm) shows the color change from orange to violet for the same angle change (the right sample of Fig. 2c). The angle-dependent color is anticipated with a model previously developed for curved interfaces^27^. However, the angle dependencies are fairly matched with models for the CRs of 12 μm and 9.5 μm rather than the measured values of 15 μm and 12.5 μm, respectively (Fig. 2b). Also, the retroreflection spectra experimentally measured are consistent with those calculated from the models with the CRs of 12 μm and 9.5 μm (Supplementary Fig. 7). This discrepancy is probably caused by the refraction of light at the weakly concave surface, which is not considered in the models (Supplementary Fig. 8). The axisymmetric geometry of micro-crescents renders the structural colors invariant for the change of observation angle along the azimuthal direction as long as the incident light is perpendicular to the suspension (Fig. 2b). However, the colors vary along the azimuthal angle of observation for the off-perpendicular illumination (Supplementary Fig. 9).

The micro-crescents exhibit conspicuous structural colors at retroreflection conditions, which remain unchanged for the variation in the observation angle from 0° to 75° (Fig. 2d and Supplementary Movie 1). It is also noteworthy that distinct colors are still observed at an angle as high as 75°. The angle-independent color is attributed to the spherical symmetry of the convex surface which allows TIR and interference along a constant curvature. Although the length of the arch of the convex surface responsible for the TIR decreases along with the angle, the refraction of light at the concave surface significantly alleviates the decrease (Supplementary Fig. 10). For example, the half cone angle of the arch at the observation angle of 75° is 53° without the refraction, which is smaller than critical angle *α* and therefore incapable of TIR. However, the refraction increases the half cone angle to 74° which is much greater than *α* and not much reduced from 81° for the normal observation (Fig. 2e). We further confirm the constant retroreflection colors for a wide range of angles by directly observing tilted micro-crescents with an optical microscope (Fig. 2f). It should be noted that two bright color spots are closely positioned to the centerline of the micro-crescent for large tilting angles of 60° and 75° due to the refraction. That is, the weak reduction of the half cone angle by refraction provides invariant structural colors for a wide range of angles at the retroreflection, which is a unique property of micro-crescents in a particle format. The reflection spectra also evidence the invariance of the retroreflection colors (Fig. 2g). The wavelengths of the primary peaks remain unchanged as the angle increases from 0° to 75°, while the intensities decrease due to the reduction of the reflection area.

### Influence of dimension and geometry on optical properties

**Fig. 3:**
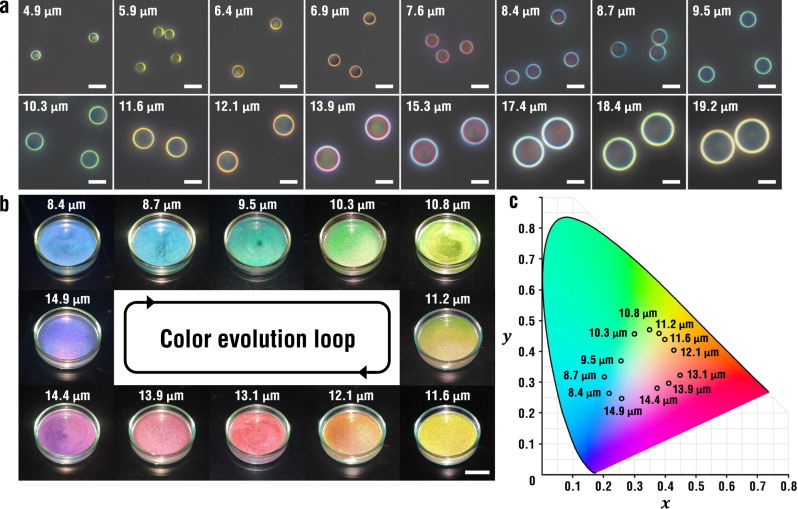
Influence of size on structural colors of micro-crescents. **a** Series of reflection optical micrographs of the micro-crescents with the radii from 4.9 to 19.2 μm. Scale bar, 20 μm. **b** One round for color cycling in the radius range of 8.4–14.9 μm in 12 steps. Illumination and observation direction are both maintained at a polar angle of 20°. Scale bar, 20 mm. **c** CIE diagram showing the circulatory shift of the coordinates for the size change in (**b**). Source data for **c** is provided as a Source Data file.

The radius of the micro-crescents is tunable by modifying the initial size of emulsion droplets or the concentration of PS and silicone oil while maintaining the identical geometry. It is conceivable that all TIR trajectories are enlarged on a larger convex surface. Longer propagation of lights inside the micro-crescent causes greater optical path differences for mutual interference, which increases the wavelengths of reflection peaks and determines the structural color. Therefore, the color change, or equivalently reflectance peak shift in the visible range, is not random yet periodic red-shift in the radius range of 4.9–19.2 μm (Fig. 3a and Supplementary Fig. 11). In a single period, various colors can be developed by finely tuning the radius of micro-crescents. For example, a gradual color shift from blue to cyan, green, yellow, orange, red, magenta, and purple in the full visible range is demonstrated with 12 steps in the radius range of 8.4–14.9 μm (Fig. 3b), which makes a complete loop in the chromaticity diagram (Fig. 3c). When the radius of the micro-crescents is greater than 50 μm, a bright ring is observed in each micro-crescent but no color is developed (Supplementary Fig. 12). Although the convex surface provides TIR, there are many pairs of rays are available for optical interference, which results in multiple reflection peaks in the visible range, rendering the micro-crescents colorless.

**Fig. 4:**
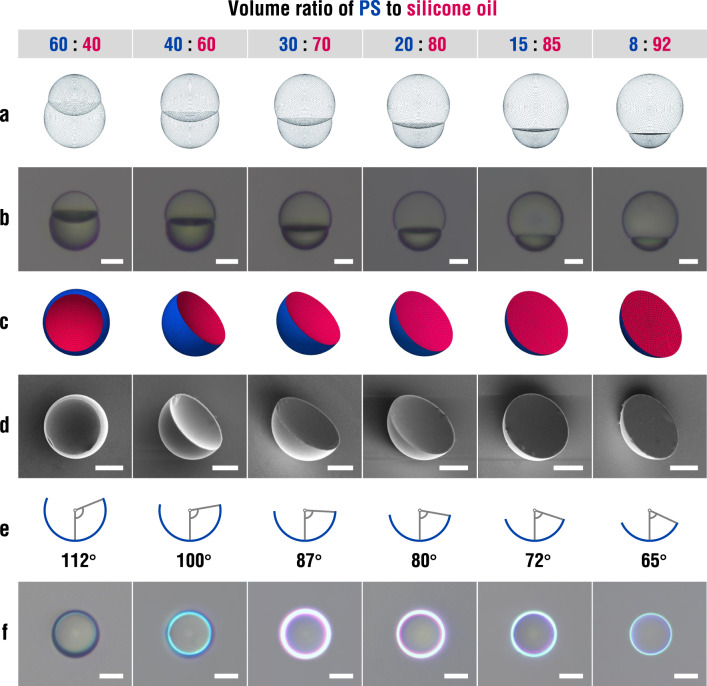
Control of micro-crescent geometry. **a, b** Sets of *Surface Evolver* model and reflection optical micrograph of paired microparticles with volume ratios of PS to silicone oil as denoted. Scale bar, 20 μm. **c, d** *Surface Evolver* model and SEM image of micro-crescents. Scale bar, 20 μm. **e, f** Cross-sectional view with geometric angle and reflection optical micrographs with structural colors of the micro-crescents. Scale bar, 20 μm.

The structural colors are also affected by the geometry of the micro-crescents, which is mainly represented by the half cone angle of the convex surface relative to the basal plane. This geometric angle is controllable by adjusting the relative volume ratio of PS relative to silicone oil. As the volume ratio changes from 60: 40 to 8: 92, the relative size of the PS compartment decreases, and the geometric angle for the convex surface significantly decreases (Fig. 4b). The shapes of the paired microparticles are consistent with *Surface Evolver* simulation for all the volume ratios, further confirming that the shape is predominantly determined by the minimum surface energy state (Fig. 4a). The micro-crescents with various geometric angles are produced by the removal of silicone oil blobs, of which shapes are also in good accord with the *Surface Evolver* simulation (Fig. 4c, d).

To study the influence of the geometric angle on the optical property, the micro-crescents with various geometric angles yet comparable CR of approximately 20 μm are selected to compare the annular reflection (Fig. 4e, f). The brightest reflection is observed when the geometric angle is close to 90° as the number of pairs for the TIR and interference is maximized. For the geometric angles of 87° and 80°, thick blue and red rings are overlapped. As the geometric angle decreases to 72°, the thick blue ring survives while the red ring almost diminishes due to the exclusion of outermost paths of light. Further, the blue ring gets thin at 65° and the color ring is no longer revealed at 60° due to the continuous exclusion until no interfering pair is available (Supplementary Fig. 13). Although the minimum geometric angle to produce color (include two beam paths: *m* = 3 and 4) is 67.5° in theory, light from the objective lens is far from the perfect planar wave, which develops two paths even at 65°. There is a similar trend for the geometric angles greater than 90°. The rays incident at the outermost part of the periphery will be greatly refracted due to the semi-open shape of the convex surface, which are incapable of TIR and interference. Therefore, the annular reflections likewise exclude the red ring and get thinner at the angle of 100° and 112° and no color is developed at 123°. Such symmetrical color change along the geometric angle from 90° is also evidenced by the model (Supplementary Fig. 14). It should be noticed that the micro-crescents with high geometric angles show random orientations due to more spherical morphology than those with low geometric angles, which makes the structural colors less pronounced (Supplementary Fig. 15).

As determined by the minimum interfacial energy states, the shape is further tunable by changing the interfacial tensions. For example, the use of sodium dodecyl sulfate (SDS) instead of PVA as the surfactant alters the interfacial tensions for PS-water and silicone oil-water interfaces, resulting in the PS-rich compartment with strongly and weakly convex surfaces, agreeing with the *Surface Evolver* simulation with measured interfacial tensions for SDS. (Supplementary Fig. 16). The lens-shaped microparticles also show structural colors along the periphery in a similar manner to micro-crescents when weakly convex surfaces face up (Supplementary Fig. 17).

### Multicolor graphics visualized by directional lighting

**Fig. 5:**
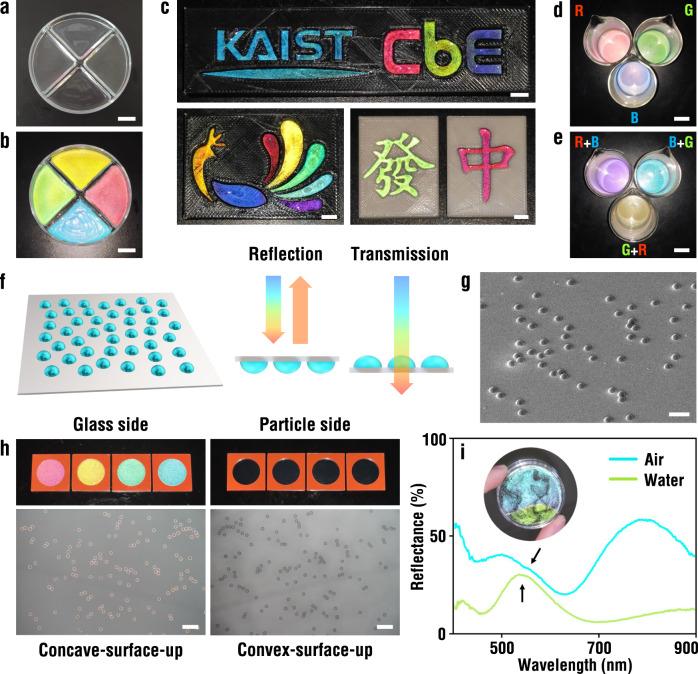
Multicolor graphics visualized by directional lighting. Photographs of aqueous suspensions of four distinct micro-crescents under ambient light (**a**) and directional light (**b**). Scale bar, 20 mm. **c** Photographs of the colorful graphics of the logo of the school and department and phoenix on a black background, and the Chinese mahjong patterns on a white background. Scale bar, 5 mm. Photograph of blue, green, and red suspensions of unary micro-crescents (**d**) and yellow, cyan, and purple suspensions of binary mixtures as denoted (**e**). Scale bar, 20 mm. **f, g** Cartoon and SEM image showing the unified orientation of micro-crescents to have convex surfaces up for one-side coloration. Scale bar, 50 μm. **h** Photographs showing the one-side coloration for the panels containing micro-crescents with radii of 20.3 μm (red), 19.2 μm (yellow), 18.4 μm (green), and 17.4 μm (cyan), and reflection optical micrographs of the red panel taken from two sides of the identical spot. Scale bar, 200 μm. **i** Reflectance spectra of dried and water-immersed micro-crescents with a radius of 10.8 μm. Inset is a photograph of the panel with two regions. Source data for **i** is provided as a Source Data file.

The micro-crescents show vivid structural colors under directional white lights at retroreflection conditions, while being transparent with negligible coloration under ambient light due to insufficient intensity of light for the TIR and interference (Fig. 5a, b and Supplementary Fig. 18). This light-selective coloration of the micro-crescents is highly beneficial as invisible inks for encoding of color graphics in various anti-counterfeiting purposes. For example, the aqueous suspensions of micro-crescents are used as invisible color inks to compose multicolor graphics by filling them in engraved patterns of a phoenix and the logo of the school and department, where multiple colors originate from different radii of the micro-crescents (Fig. 5c). The graphics are colorless under ambient light, which display vivid colors under a flashlight from a smartphone (Supplementary Movie 2). That is, the multicolor graphics are selectively visualized by the directional light. It is not necessary to use a black background for vivid coloration. The suspensions in white engraved patterns of Chinese mahjong also show high brightness and saturation of structural colors under directional light. Also, it is possible to formulate the invisible inks by suspending the micro-crescent into a proper medium to satisfy rheological conditions. For example, we use poly(ethylene glycol) (PEG) suspensions as inks and write texts by hand-writing (Supplementary Fig. 19).

The micro-crescents with different radii can be simultaneously suspended to develop mixed colors. For example, a mixture of blue and green micro-crescents produces a cyan color for a macroscopic view while individual blue and green rings are disclosed in the microscopic view (Supplementary Fig. 20). In the same manner, the binary blend of red and blue yields a violet color and that of green and red develops a yellow color, which are consistent with the superposition of tricolors (Fig. 5d, e). It is possible to finely tune the colors by adjusting the mixing ratio. The mixtures of cyan- and red-colored micro-crescent display green for the ratio of 3:1, yellow for 2:2, and orange for 1:3 (Supplementary Fig. 21). The mixing strategy is feasible to enrich the library of structural colors, like a palette.

The orientation of the micro-crescents is almost bimodal under the action of gravity, either convex-surface up (concave-surface down) or concave-surface up (convex-surface down). Therefore, about half of the micro-crescents contribute to the coloration for the one-side observation. It is possible to unify the orientation of the micro-crescents to be concave-surface down on the substrate by repeating the sinking and shaking. The micro-crescents with concave-surface down are far more sedentary on the substrate than those with convex surface down. The micro-crescents with convex-surface down are re-suspended in water by gentle shaking, which submerges to have two different orientations again. Therefore, several steps of sinking and shaking make most of the micro-crescents concave-surface down (Fig. 5f, g). When employing a transparent substrate, the panel exhibits Janus optical property (Fig. 5h and Supplementary Movie 3). Brilliant colors are only observed from the back side of the panel as the convex surface of the micro-crescents is available for the TIR and interference of the incident light. By contrast, the same panels are transparent and show no color when observed from the front side where the micro-crescents are present.

The surrounding of the micro-crescents is also critical for coloration^45^. For example, the micro-crescent array displays real-time color contrast between the segments submerged in water (red) and exposed in air (blue) (Supplementary Movie 4). This color variation is caused by the different refractive index of the medium, which changes the value of *α* for TIR from *α* = 56.8° in the water to *α* = 39° in the air. Therefore, more interfering pairs of rays contribute to the reflection in the air even though the curvature is the same, resulting in different reflective colors. As shown in Fig. 5i, the reflectance spectrum measured from the cyan-colored micro-crescents in air shows multiple peaks including the main resonances at 400, 490, and 800 nm, whereas that from the array in water shows one main peak at 540 nm, which is responsible for green color. It is noteworthy that the spectrum for the air medium also has a small peak around 540 nm possibly originating from the same trajectory to the water medium, which is overwhelmed by other resonance. To study the influence of the refractive index of the surrounding systematically, we employ various media with different refractive indexes (Supplementary Fig. S22). As the refractive index of the medium increases toward that of PS, the number of available beam paths guided by TIR decreases, which results in color brightness reduction. The hue is fairly well predicted with the model.

## Discussion

Structural coloration is promising for various applications, including reflection-mode displays, colorimetric sensors, and anti-counterfeiting materials. Nevertheless, the practical fabrication of periodic structures at a sub-wavelength scale with photonic bandgaps is somewhat limited due to high cost, low throughput, and delicate conditions. Alternatively, the structural colors can be developed by TIR and optical interference with curved interfaces in the absence of complicated periodic structures, which provides relatively simple and facile fabrication schemes. Although there are many differences in the optical properties, it is possible to replace photonic bandgap structures with curved interfaces in certain applications. To pragmatically use the curved interfaces, the structures should be implemented in a form of inks and directly applied on arbitrary target surfaces rather than multiple replications of surfaces. To achieve this goal, we have designed the micro-crescents as full-color reflective pigments that are suspendable in liquid media to formulate inks. As the convex surfaces are pre-defined in the micro-crescents, stable colors are developed and the refraction at a weakly concave surface provides a wide viewing angle for invariant color at retroreflection. Moreover, the inks can be applied to target surfaces to directly develop the structural colors. As the colors are invisible under ambient light yet highly visible and brilliant under directional light, the micro-crescents are particularly appealing for anti-counterfeiting patches, color cosmetics, and color coatings with distinctive impressions.

The nanostructure-free micro-crescents are suitable for scalable production, due to their highly-efficient manufacturing process of emulsion templating and substantialy low cost of raw materials. As the micro-crescents are produced by spontaneous phase separation in single emulsion droplets rather than direct use of biphasic droplets, it is more feasible for the scaleup using 3D-printed or parallelized droplet makers^46–51^. For example, millipede microfluidic devices can be used to integrate more than 500 droplet makers, which can significantly improve the production throughput^52^. Although size uniformity should be guaranteed for uniform coloration, various emulsification techniques, including controlled membrane emulsification, can be used in a conjunction with size-dependent fractionation techniques for mass production. Moreover, the set of materials for emulsion templating is not limited to PS and silicone oil. For example, polymethyl methacrylate (PMMA) (*n* = 1.49) and poly(propylene carbonate) (PPC) (*n* = 1.58) can be used as a polymer to compose the micro-crescents instead of PS and soybean oil can replace silicone oil as a sacrificial blob (Supplementary Fig. 23). There is room to further develop the micro-crescent for advanced applications. For example, the micro-crescents can be magnetically functionalized for real-time control of their orientation for structural color switching (Supplementary Movie 5). We expect our findings will facilitate the practical uses of TIR-interference-based structural colors in our daily lives.

## Methods

### Materials

Polystyrene (PS, average *M*_w_ = 35 kg mol^−1^), poly(methyl methacrylate) (PMMA, average *M*_w_ = 2 kg mol^−1^), poly(ethylene glycol) (PEG, average *M*_w_ = 200 g mol^−1^), poly(propylene carbonate) (PPC, average *M*_w_ = 50 kg mol^−1^), poly(vinyl alcohol) (PVA, average *M*_w_ = 13–23 kg mol^−1^, 87–89% hydrolyzed), silicone oil (viscosity 5 cSt), soybean oil, chloroform (purity > 99%) were all purchased from Sigma-Aldrich. 2[methoxy(polyethyleneoxy)propyl]trimethoxy silane was purchased from Gelest, Inc. Deionized water (Millipore Milli-Q grade) with a resistivity of 18.0 MΩ was used in all the experiments.

### Preparation of micro-crescents

PS (10 mg) and silicone oil (30 mg) are dissolved in chloroform (2.7 mL) and then emulsified by PVA aqueous solution (10% w/w). Monodispersed chloroform single emulsion droplets were prepared by a capillary microfluidic device. The device is composed of two tapered cylindrical capillaries (inner diameter 0.58 mm, outer diameter 1.0 mm, World Precision Instruments) and a square glass capillary (inner diameter 1.05 mm, outer diameter 1.50 mm, Atlantic International Technologies, Inc.). One capillary was sanded to a 60 μm orifice, termed the injecting capillary. The other was sanded to a 120 μm orifice and then treated with 2[methoxy(polyethyleneoxy)propyl]trimethoxy silane for 30 min at 50 °C to render the surfaces hydrophilic, termed outlet capillary. Two cylindrical capillaries were tip-to-tip coaxially aligned within the square capillary with a distance of 80 μm. The chloroform solution was injected through the injection capillary as the inner phase, while PVA aqueous solution was injected from the same direction through the interstice between the injection capillary and the square capillary as the continuous phase. The inner flow rate was set from 200 to 400 μL h^−1^ and outer flow rates were typically set from 1000 to 3000 μL h^−1^. The droplets were collected from the outlet capillary and incubated in PVA aqueous solution (1% w/w) at 40 °C to evaporate chloroform. During the incubation for 1 day, the droplets were transformed into biphasic ones, where PS was completely consolidated. The droplets were subjected to ultrasonication at 60 °C for 20 min to remove silicone oil blobs from the consolidated PS micro-crescents. Finally, the micro-crescents were separated by centrifugation (500 × *g*) and then washed with distilled water several times.

### Characterization

The microfluidic fabrication of emulsion droplets was monitored by an optical microscope (OM, Eclipse TS100, Nikon) equipped with a high-speed camera (MotionScope M3, Redlake). The shape of microparticles was characterized with a scanning electron microscope (SEM, S-4800, Hitachi). Micro-crescents are observed using an OM (Eclipse L150, Nikon) in reflection mode equipped with a CCD camera (DS-Ri2, Nikon). The reflectance spectra are measured using a fiber-coupled spectrometer (HR4000CG-UV-NIR, Ocean Optics) installed in the OM.

### Simulation

The hemisphere models for the color distribution of the micro-crescents are implemented by MATLAB. By imputing the parameters for the RI of two mediums *n*_1_ and *n*_2_, the light incidence angle *θ*, curvature radius *R*, and the half cone angle *η* of the curved interface, each value of outgoing angle *θ*_out_ and wavelength can be summarized as a model. The MATLAB code is downloaded from https://github.com/snnagel/Structural-color-by-Cascading-TIR/Create color distribution^27^.

### Reporting summary

Further information on research design is available in the Nature Portfolio Reporting Summary linked to this article.

## Supplementary information

Supplementary Information File

Description of Additional Supplementary Information

Supplementary Movie 1

Supplementary Movie 2

Supplementary Movie 3

Supplementary Movie 4

Supplementary Movie 5

Reporting Summary

## Data Availability

The authors declare that the main data supporting the findings of this study are available within the article and its Supplementary Information files. Extra data are available from the corresponding author upon request. Source data are provided with this paper.

## Source data

Source Data
