# Supplementary material for: Nanostructure-free crescent-shaped microparticles as full-color reflective pigments
Source: Nat Commun. 2023 Feb 11;14:793. doi: 10.1038/s41467-023-36482-4 (PMC9922275; doi:10.1038/s41467-023-36482-4)
Supplement: Supplementary file 1 — Supplementary Information File [file 41467_2023_36482_MOESM1_ESM.pdf]

Supplementary Information

Nanostructure-Free Crescent-Shaped Microparticles as Full-Color Reflective Pigments

Yi Yang[1,2], Jong Bin Kim[1], Seong Kyeong Nam[1], Mengmeng Zhang[2], Jiangping Xu[2], Jintao Zhu[2]*, and Shin-Hyun Kim[1]*

[1]Department of Chemical and Biomolecular Engineering, Korea Advanced Institute of Science and Technology (KAIST), Daejeon 34141, Korea

[2]Key Laboratory of Material Chemistry for Energy Conversion and Storage, Ministry of Education, School of Chemistry and Chemical Engineering, Huazhong University of Science and Technology (HUST), Wuhan 430074, China

* Correspondence: kim.sh@kaist.ac.kr and jtzhu@hust.edu.cn

Supplementary Figures

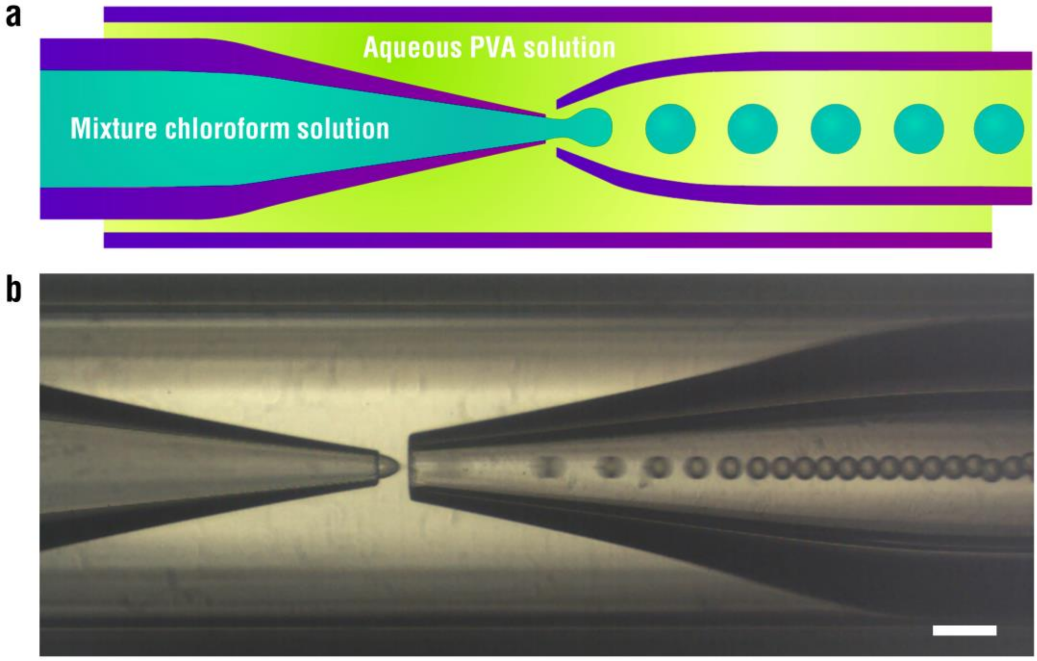

Supplementary Fig. 1 | Microfluidic production of monodisperse emulsion droplets. Schematic **(a)** and optical micrograph **(b)** demonstrate that a capillary microfluidic device is used to prepare monodisperse oil-in-water emulsion droplets. Scale bar, 200 μm. The orifices of the injecting and collecting capillaries are 60 and 120 μm, respectively. The inner phase is chloroform solution of polystyrene (PS) and silicone oil, and the continuous phase is aqueous solution of poly(vinyl alcohol) (PVA). The flow rates are set as 250 μL h^{-1} for the inner phase and 3500 μL h^{-1} for the continuous phase.

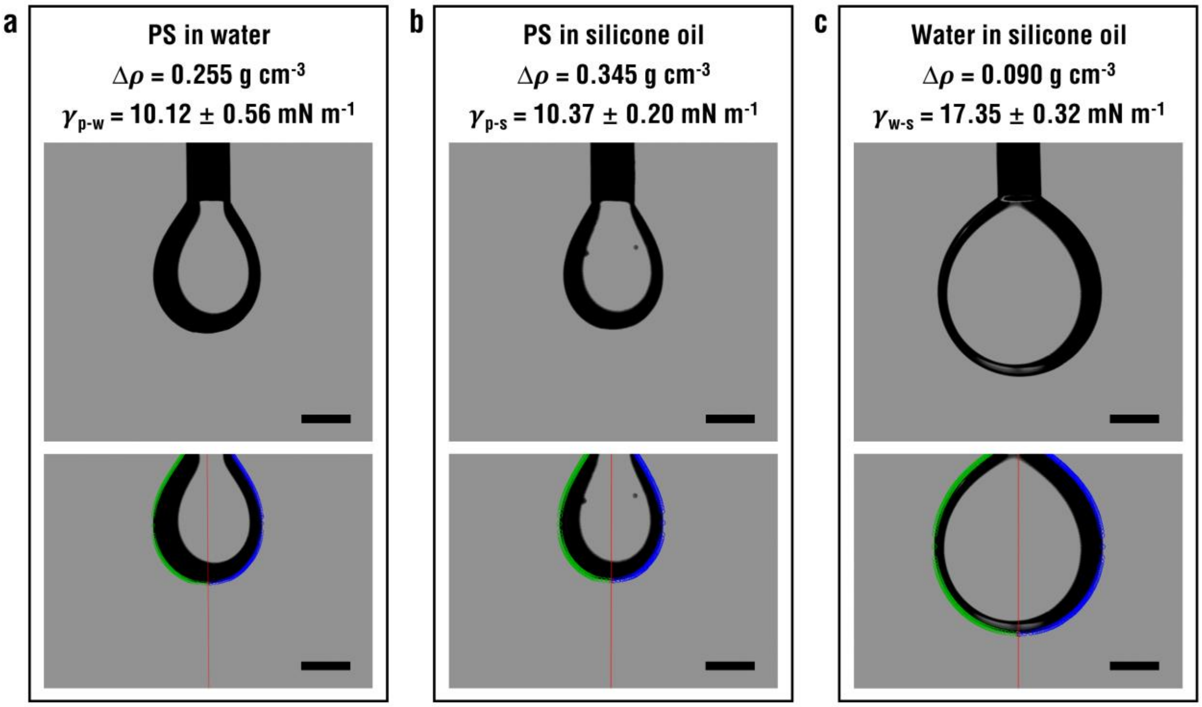

Supplementary Fig. 2 | Measurement of interfacial tensions. The interfacial tensions of PS-to-water ($\gamma_{p\text{-}w}$), PS-to-silicone oil ($\gamma_{p\text{-}s}$), and water-to-silicone oil ($\gamma_{w\text{-}s}$) are measured using a pendant drop method. For PS phase, we use a chloroform solution of PS at the concentration of 48% w/w. The density of the PS-chloroform solution, silicone oil, and water (PVA solution) is 1.258, 0.913, and 1.003 g cm^{-3}, respectively. We produce a pendant drop of heavier phase into a lighter phase. The tensions are measured as $\gamma_{p\text{-}w}$ = 10.12 mN m^{-1} **(a)**, $\gamma_{p\text{-}s}$ = 10.37 mN m^{-1} **(b)**, and $\gamma_{w\text{-}s}$ = 17.35 mN m^{-1} **(c)**. Scale bar, 1 mm. The spreading parameters are negative so that a Janus structure is expected rather than a core-shell structure. The detailed shape of the paired drop is simulated using *Surface Evolver* with the measured interfacial tensions, which are highly consistent with the experimental observation.

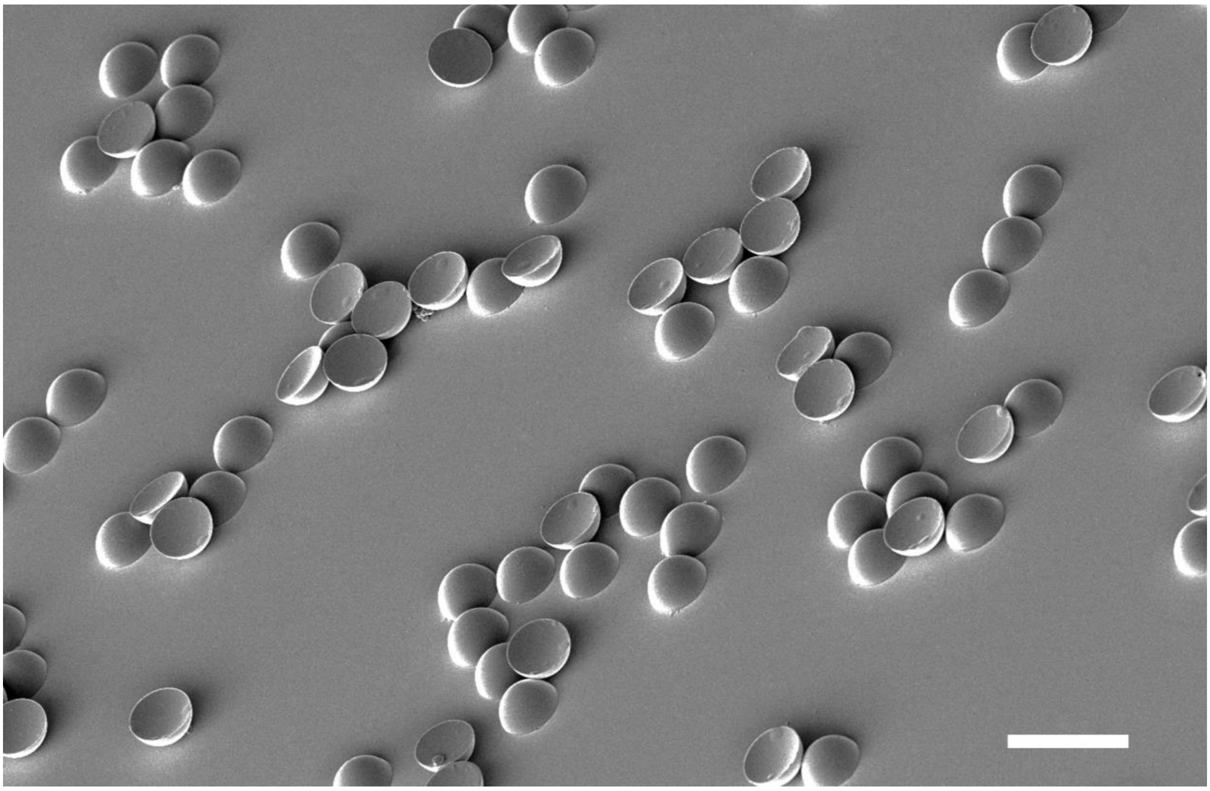

Supplementary Fig. 3 | SEM image of monodisperse micro-crescents. Scale bar, 50 μm.

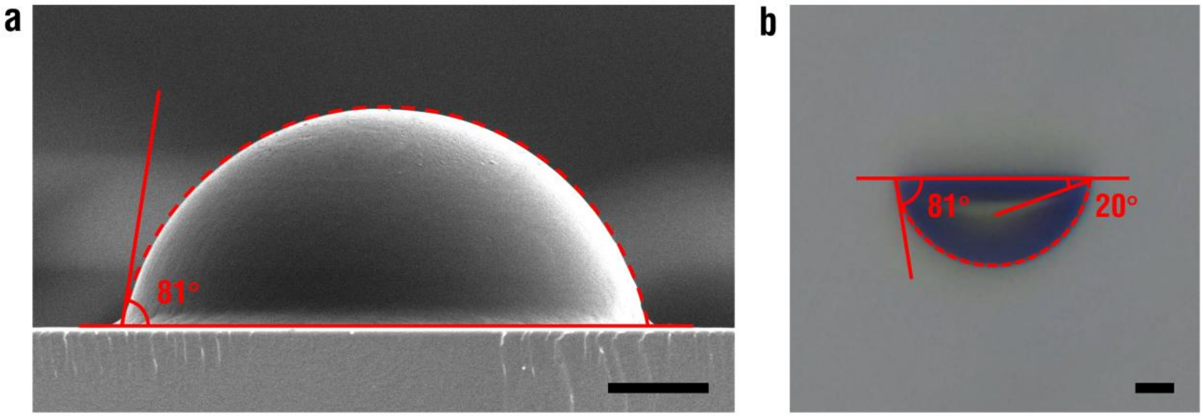

Supplementary Fig. 4 | Estimation of the geometrical angles for the convex and concave surfaces from SEM (a) and optical micrograph (b). Scale bar, 5 µm.

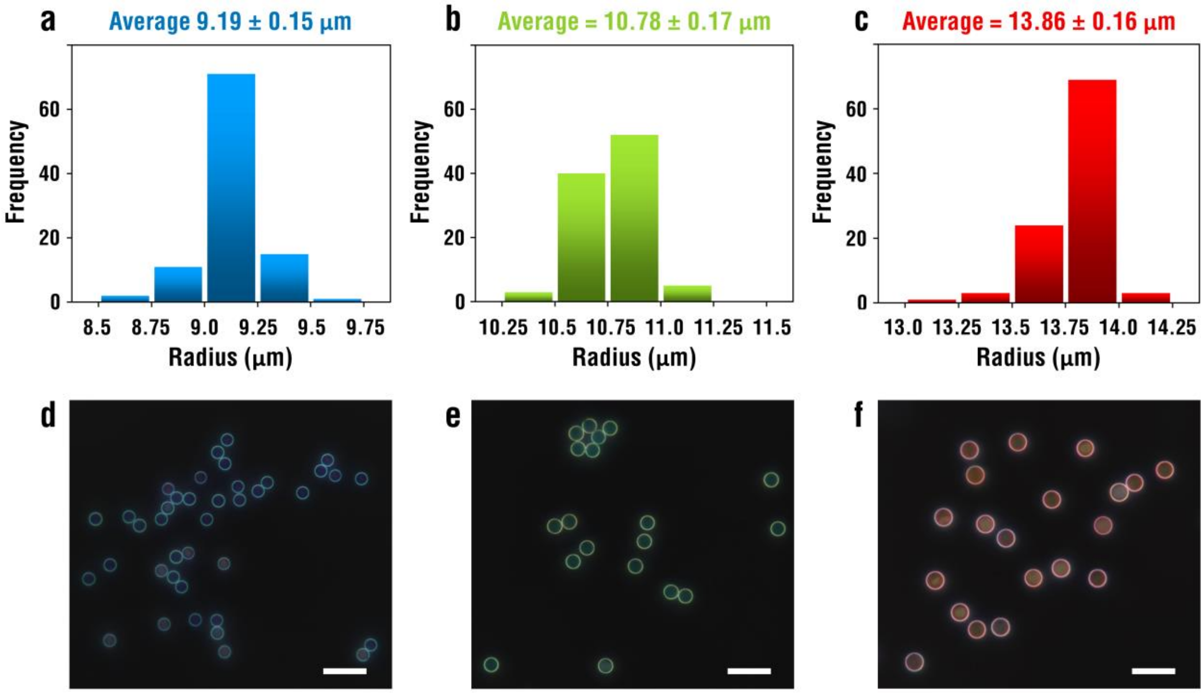

Supplementary Fig. 5 | Radius distributions with standard deviations (100 random specimen) for three micro-crescents with different radii (a–c) and corresponding reflection optical micrographs (d–f). Scale bar, 50 μm.

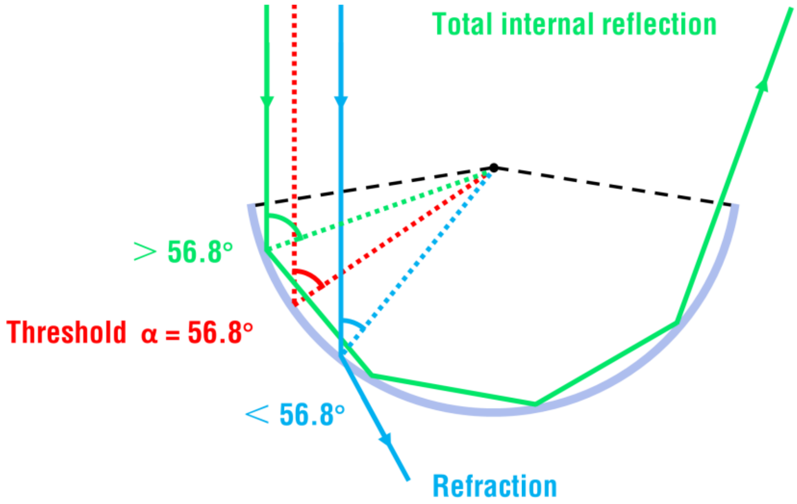

Supplementary Fig. 6 | Illustration of the critical angle for total internal reflection (TIR). The critical angle for TIR is determined by refractive index of PS (n_{PS} = 1.59) and water (n_{water} = 1.59) as $\alpha = \sin^{-1}(n_{water}/n_{PS})$ = 56.8°. Therefore, the vertically incident beam undergoes TIR on the convex surface when the incident angle is larger than 56.8° (denoted with green trajectory). Otherwise, the beam refracts at the convex surface and shows negligible reflection (denoted with blue trajectory). Therefore, structural colors are developed near the periphery of micro-crescent through the interference of beams guided by TIR.

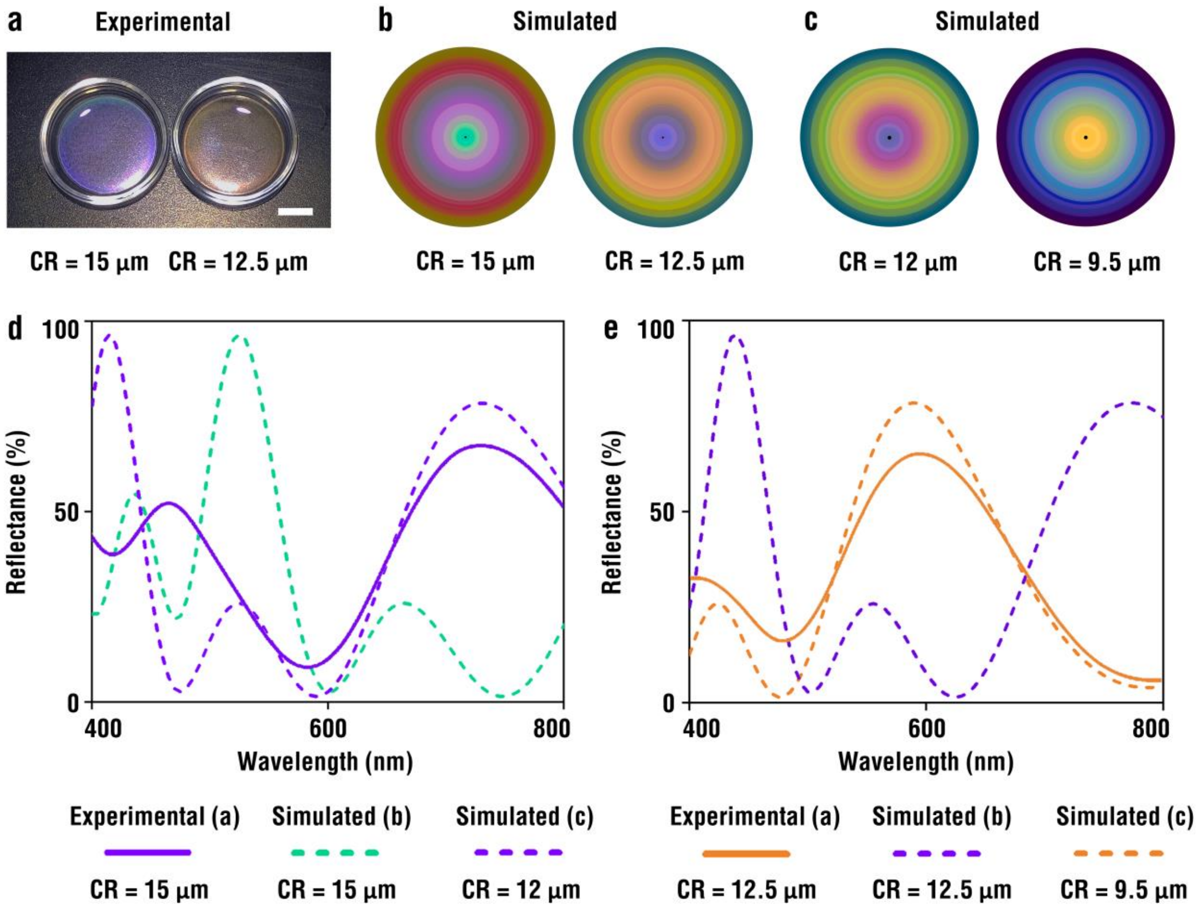

Supplementary Fig. 7 | Spectral comparison of experiment and calculation. Retroreflection spectra experimentally measured from the micro-crescents with curvature radii (CRs) of 15 μm and 12.5 μm **(a)** are matched with those from the hemispherical model with CRs of 12 μm and 9.5 μm **(c)** rather than 15 μm and 12.5 μm **(b)**, which is probably attributed to the refraction of light at the concave interface of the micro-crescents. Scale bar, 10 mm.

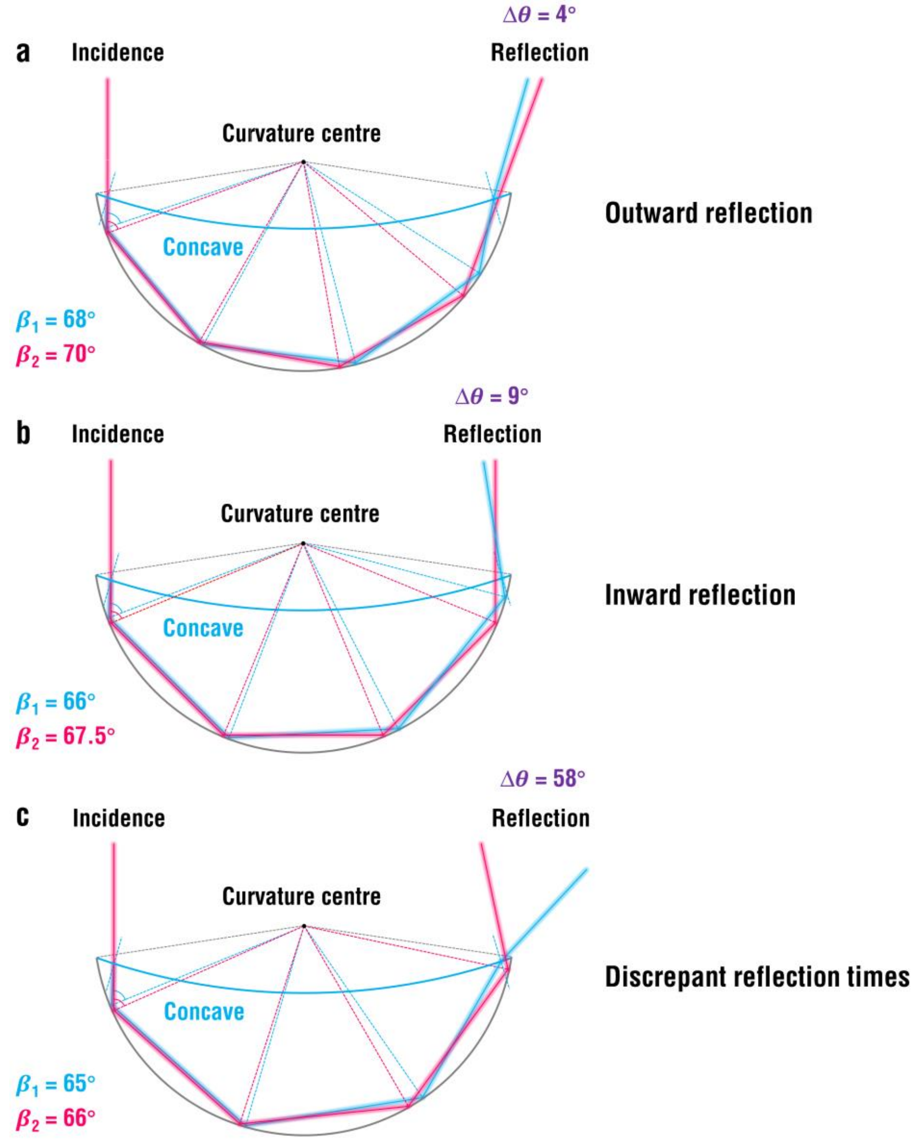

Supplementary Fig. 8 | Influence of refraction at concave surface on beam path. Beam paths of vertically incident light at three different positions near the periphery of the micro-crescent with (blue trajectories) and without (red trajectories) refraction at the concave surfaces. The refraction causes slightly smaller incident angles ($\beta_1 < \beta_2$) and the deviation get amplified as the beam propagates by multiple steps of TIR, leading to a significant difference at the outgoing angle ($\Delta\theta$) and the number of TIR.

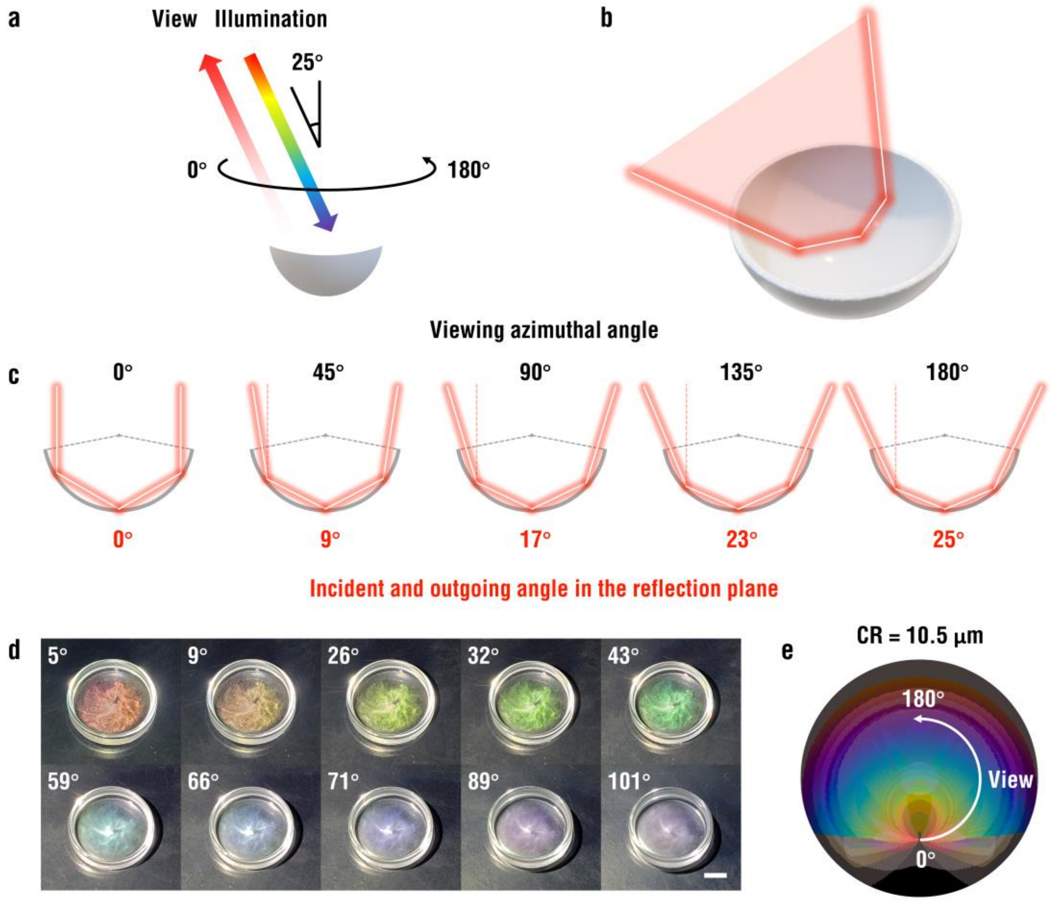

Supplementary Fig. 9 | Iridescence for off-normal illumination. (a) Under a fixed illumination with a polar angle of 25°, observation is kept at a same polar angle but with a variation of azimuthal angle. **(b)** An example of a beam trajectory responsible for TIR and interference for off-normal illumination and observation, where the trajectory lies in a plane that is perpendicular to the convex surface. **(c)** Planes of trajectory responsible for TIR and interference for various azimuthal angles between the incident beam and observation from 0° to 180°. The angles of the incident and outgoing beams remain equal due to the symmetry of the trajectories, which changes from 0° to 25°, resulting in color change. **(d)** Series of photographs showing the color change for the micro-crescents with an average radius of 13.1 μm and average CR of 13.3 μm along with the azimuthal angle, as denoted. Scale bar, 10 mm. **(e)** Top-view color map for the curved interfaces with CR of 10.5 μm under a slanted illumination at the polar angle of 25°, where the azimuthal angle is denoted with an arrow. The color map is consistent with observation.

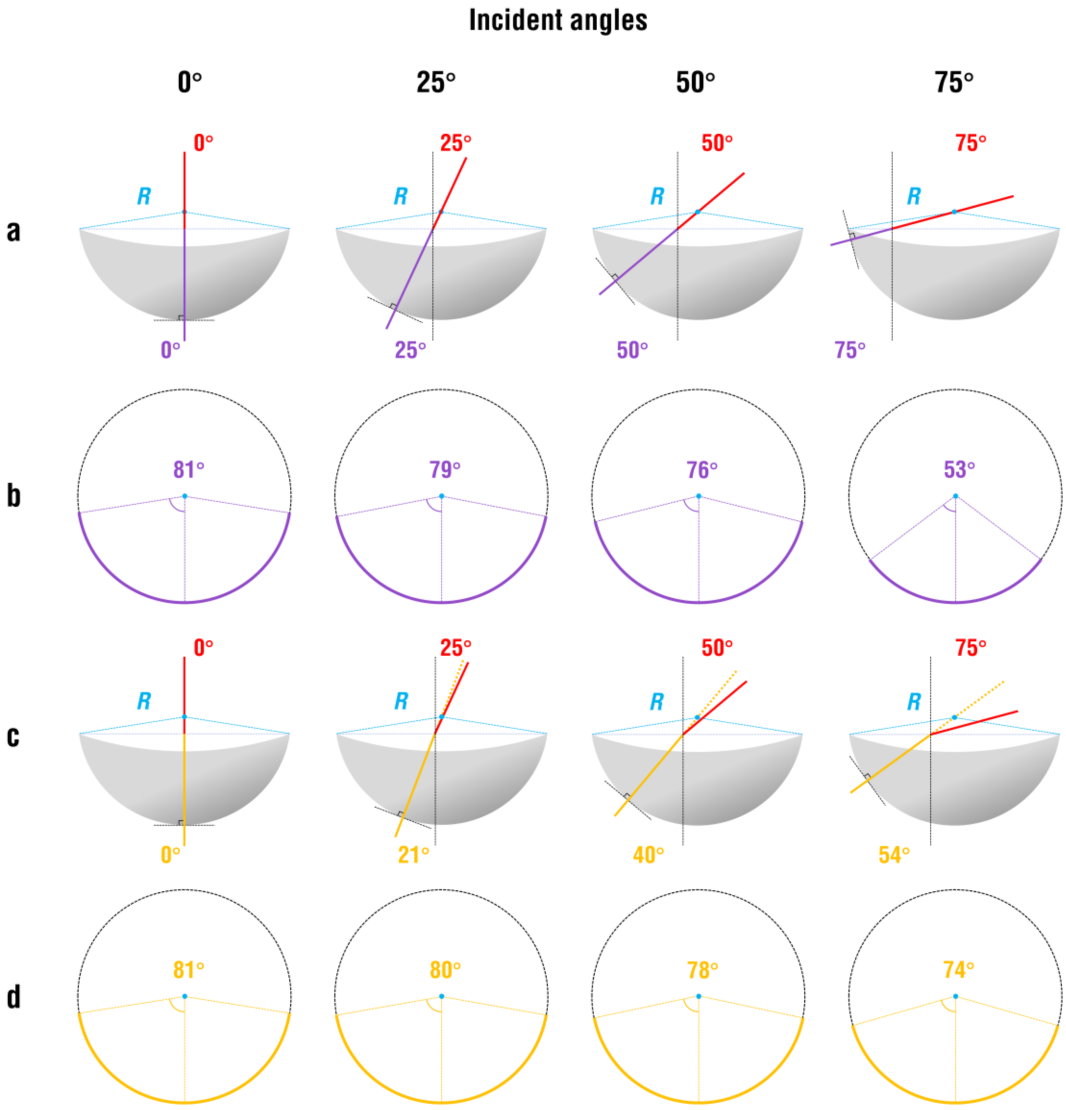

Supplementary Fig. 10 | Influence of refraction at concave surface on the half cone angle for retroreflection plane. (a, b) Cartoons for the beam paths and planes responsible for TIR at retroreflection conditions without refraction at concave surface. **(c, d)** The same set with refraction. The refraction causes a wider length of arch in the plane for TIR and larger half cone angle, in particular for large incident angles, thereby providing a consistent structural color for a wide angle of observation at retro-reflection conditions.

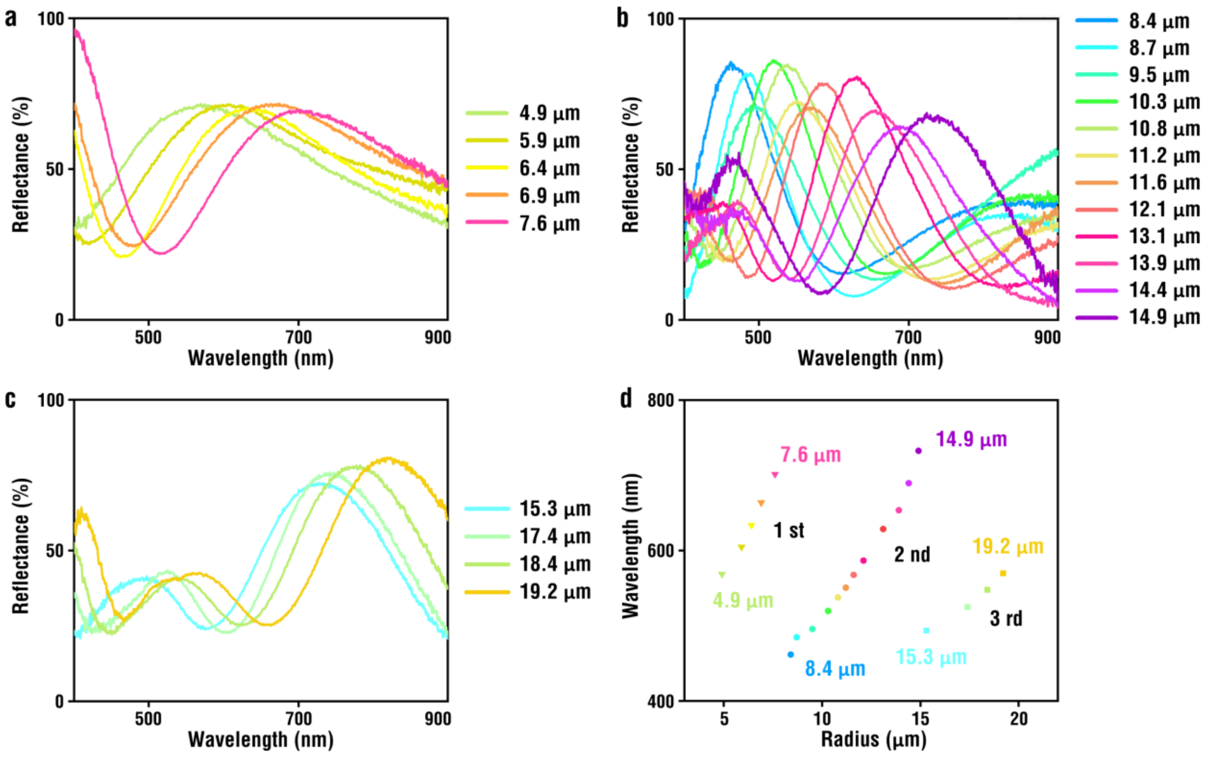

Supplementary Fig. 11 | Radius-dependent variation of retroreflection spectra. (a–c) The retroreflection spectrum red-shifts along with the radius of the micro-crescents in the entire radius range of 4.9 μm – 19.2 μm. **(d)** The main peak position within the visible range of 400 nm – 700 nm periodically red-shifts in three radius ranges of 4.9 μm – 7.6 μm, 8.4 μm – 14.9 μm, and 15.3 μm – 19.2 μm.

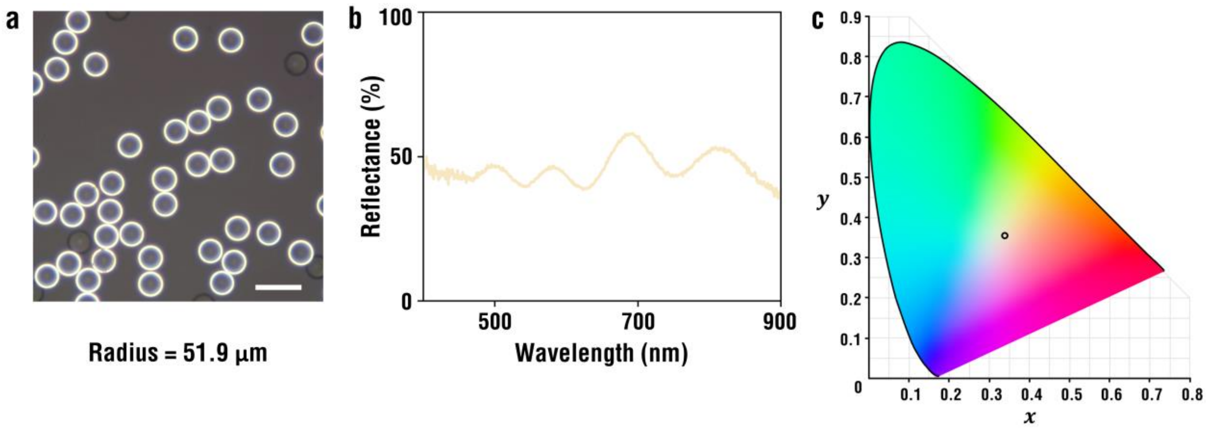

Supplementary Fig. 12 | No colors for large micro-crescents. The micro-crescents whose radius is greater than 50 μm show a bright yet colorless ring in the reflection optical micrograph **(a)** and multiple peaks in reflectance spectra **(b)** due to many sets of beam pairs for interference. The spectrum is corresponding to the coordinates of (0.344, 0.357) in the chromaticity diagram, which is almost in the white region **(c)**. Scale bar, 200 μm.

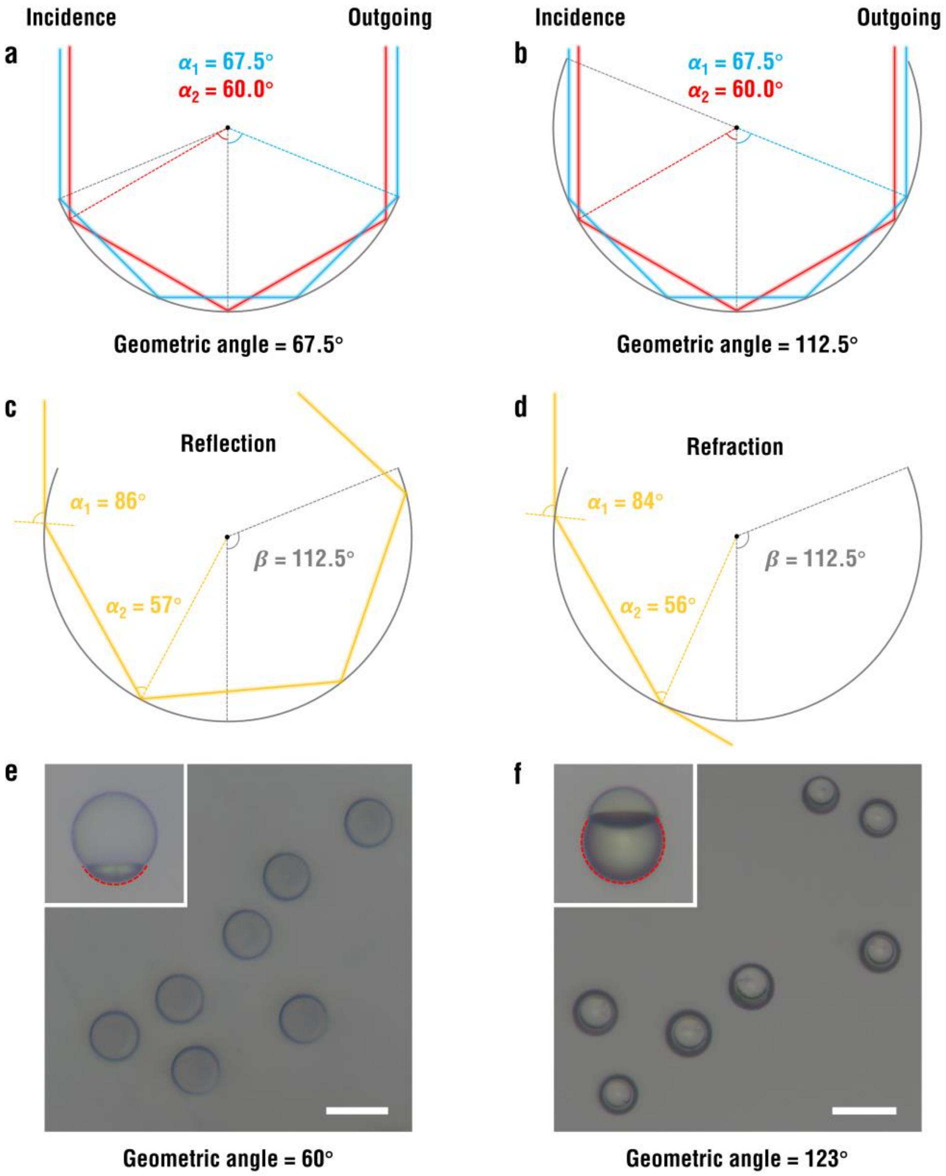

Supplementary Fig. 13 | Range of geometric angle responsible for structural coloration. (a, b) Two beam paths with triple (m = 3) and quadruple (m = 4) reflection at the convex interfaces with a geometric angle of 67.5° and 112.5° for vertical incident light, beyond which only one beam path of m = 3 is available and no color is developed. **(c, d)** Possible beam paths at the external rim of the convex interface with a geometric of 112.5°, which are finally reflected slantly or refracted. **(e, f)** Reflection optical micrograph of the micro-crescents with the geometric angles of 60° and 123°, which show no color ring. Scale bar, 50 μm.

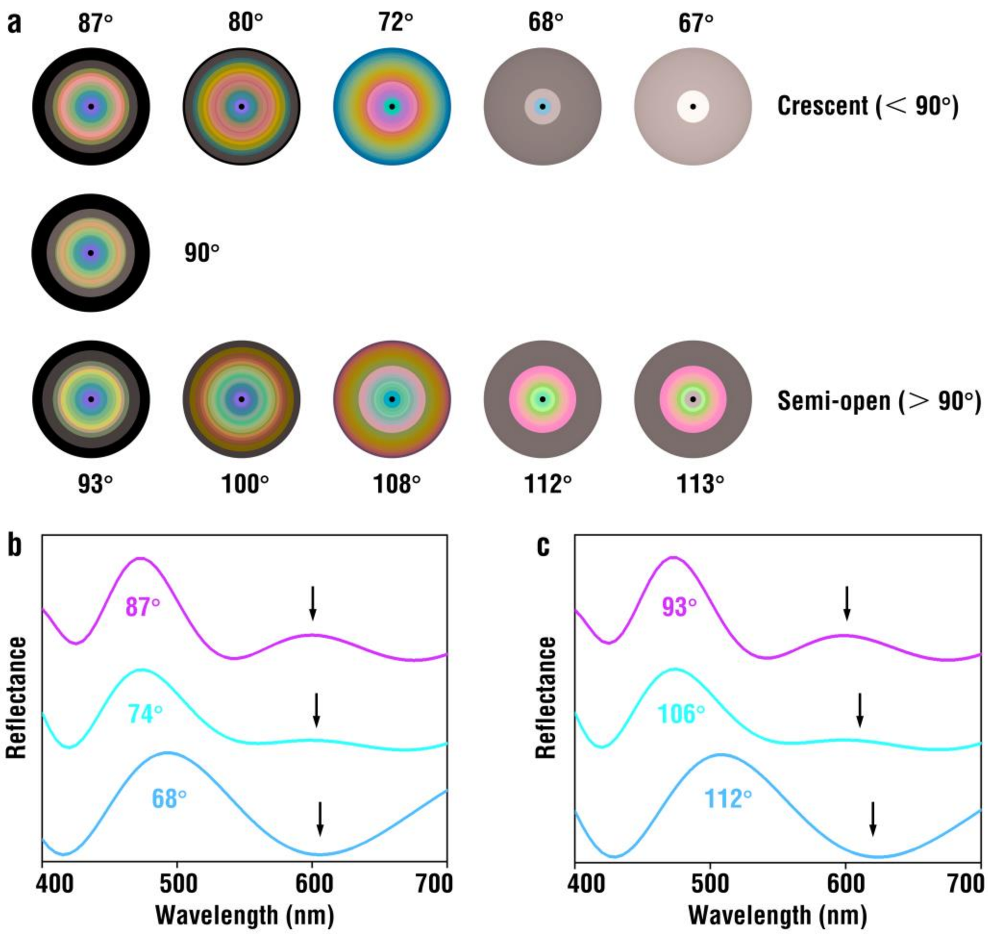

Supplementary Fig. 14 | Influence of geometric angle on color and spectrum. (a) Top-view color maps for various geometric angles in the range of 67° – 113° at a constant curvature radius of 13.5 μm calculated from the model. **(b, c)** Retroreflection spectra for the geometric angles smaller than 90° and greater than 90°, calculated from the model. No color is available for the angles of 67° and 113° in (a) as the angles are out of the range responsible for interference, which is 67.5° – 112.5°. The retroreflective color shifts from purple to blue as the geometric angle deviate from 90°. Accordingly, one peak near the wavelength of 600 nm disappears due to the exclusion of the beam path with $m > 4$ in the spectra. The trend of the color and spectrum change is consistent with Fig. 4.

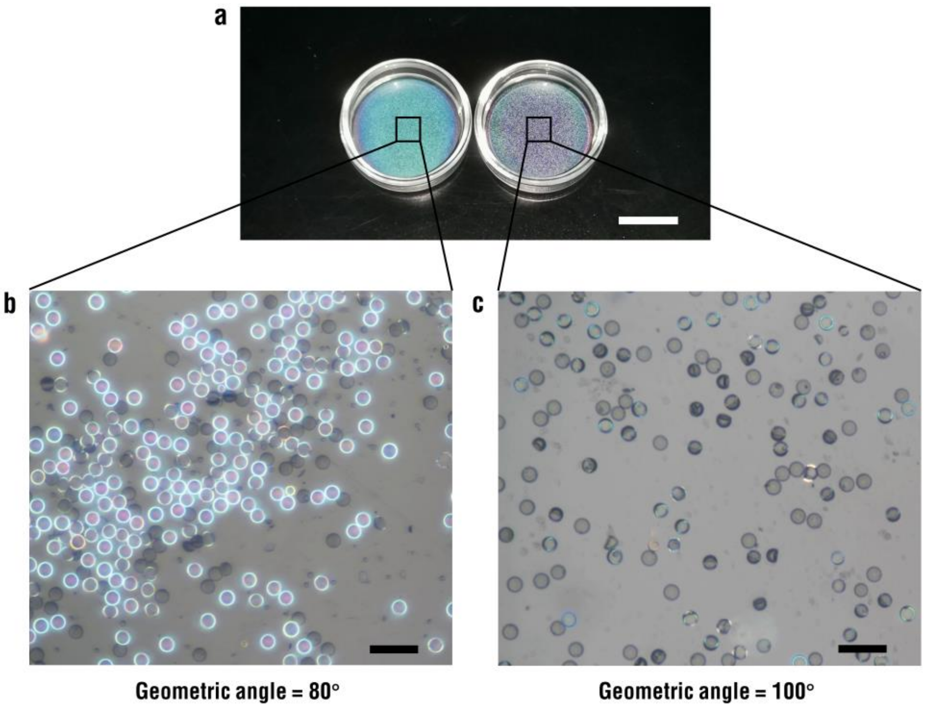

Supplementary Fig. 15 | Orientation of the micro-crescents with a geometric angle smaller and greater than 90°. (a) The photograph showing the brightness contrast between the micro-crescents with geometric angles of 80° and 100°. Scale bar, 20 mm. **(b, c)** Reflection optical micrographs disclose that the micro-crescents with a geometric angle of 80° have bi-stable modes and half of them contributes to the reflection. In contrast, the micro-crescents with a geometric angle of 100° possess more spherical morphologies, which makes them randomly oriented. Scale bar, 100 μm. Therefore, low fraction of micro-crescents shows color reflection, which reduces the brightness of colors.

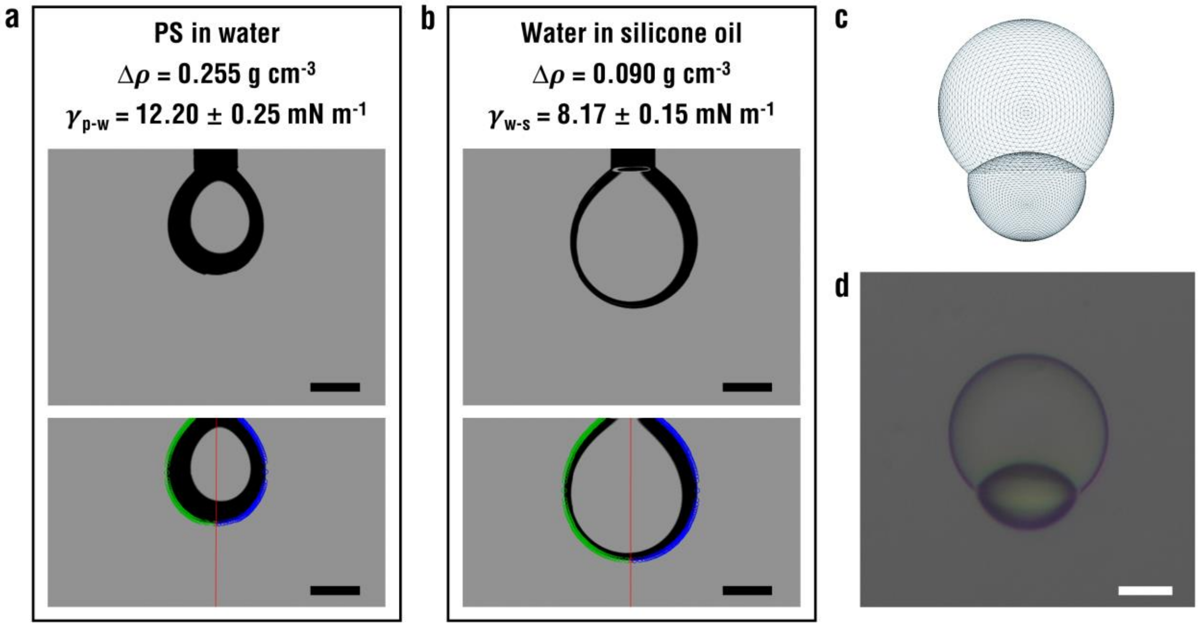

Supplementary Fig. 16 | Measurement of the interfacial tensions for SDS-stabilized interfaces. With SDS as a surfactant, instead of PVA, interfacial tensions are altered to $\gamma_{p\text{-}w}$ = 12.20 mN m^{-1} **(a)** and $\gamma_{w\text{-}s}$ = 8.17 mN m^{-1} **(b)** as measured by a pendent drop method. Scale bar, 1 mm. With the tensions, the detailed shape of paired drop is constructed by *Surface Evolver* **(c)**, which is fairly matched with experimental observation **(d)**. Scale bar, 20 μm. The resulting microparticles are lens-shaped, of which one surface is strongly convex and the other is weakly convex.

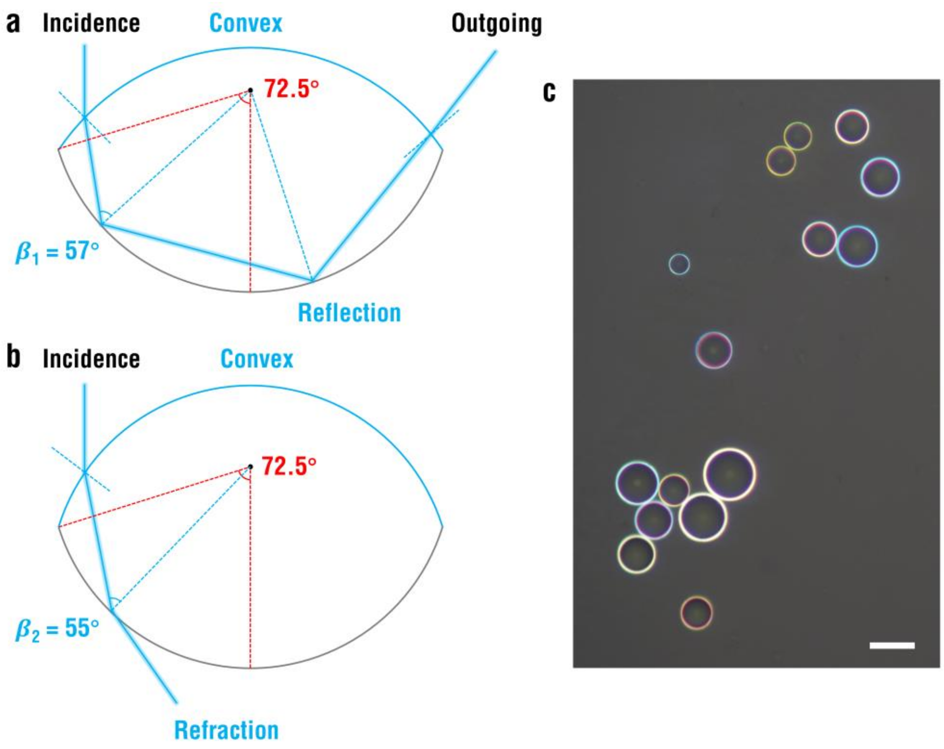

Supplementary Fig. 17 | Coloration by lens-shaped microparticles. Trajectories of vertically incident beam into lens-shaped microparticles with different convexity of top surface at the same lateral position from the center: **(a)** weakly convex top surface from the experimental observation of lens-shaped microparticles produced with SDS and **(b)** strongly convex top surface same with the bottom. The blue and purple trajectories are the optical paths with and without refraction at the top surfaces, respectively. **(c)** Reflection optical micrograph of the lens-shaped microparticles which show the color rings. Scale bar, 50 μm. The weakly convex top surface has less strong refraction at the top than the strongly convex one, which results in larger incident angle onto strongly-convex bottom surface ($\beta_1 > \beta_2$), thereby providing more beam paths for TIR and exhibiting colors. The strong refraction at the top surface prevents the TIR on the bottom surface, which is the reason why no colors are observed for spherical microparticles.

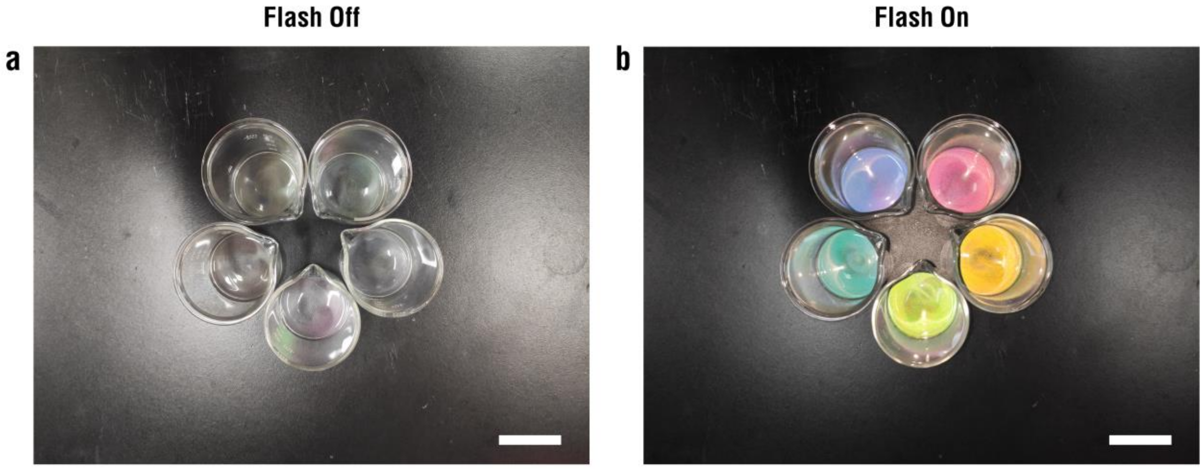

Supplementary Fig. 18 | The photographs of the five distinct suspensions of micro-crescents with different sizes under ambient light (a) and directional light (b). Scale bar, 50 mm.

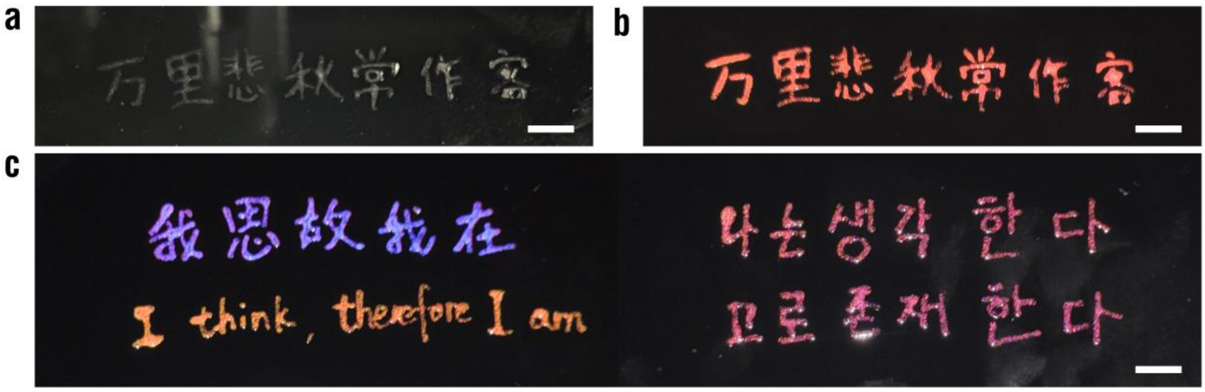

Supplementary Fig. 19 | Direct writing with inks. By employing suspensions of micro-crescent in poly(ethylene glycol) (PEG) as inks, texts are written by hand-writing. The texts show no color under ambient light **(a)** while showing pronounced structural colors under directional light **(b, c)**. Scale bar, 10 mm.

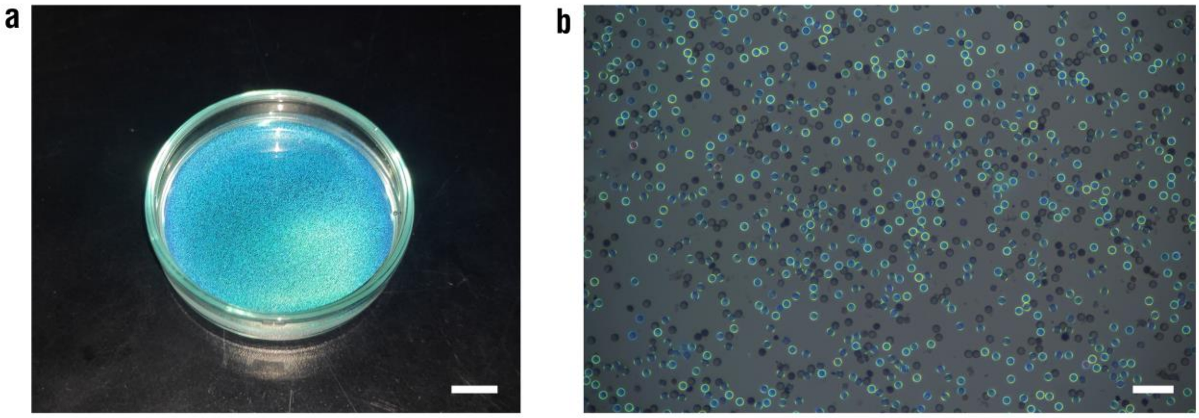

Supplementary Fig. 20 | Binary color mixing. (a) The photograph shows cyan color as a result of color mixing by the blue- and green-colored micro-crescents. Scale bar, 10 mm. **(b)** The corresponding reflection optical micrograph resolves green and blue colors of individual micro-crescents. Scale bar, 100 μm.

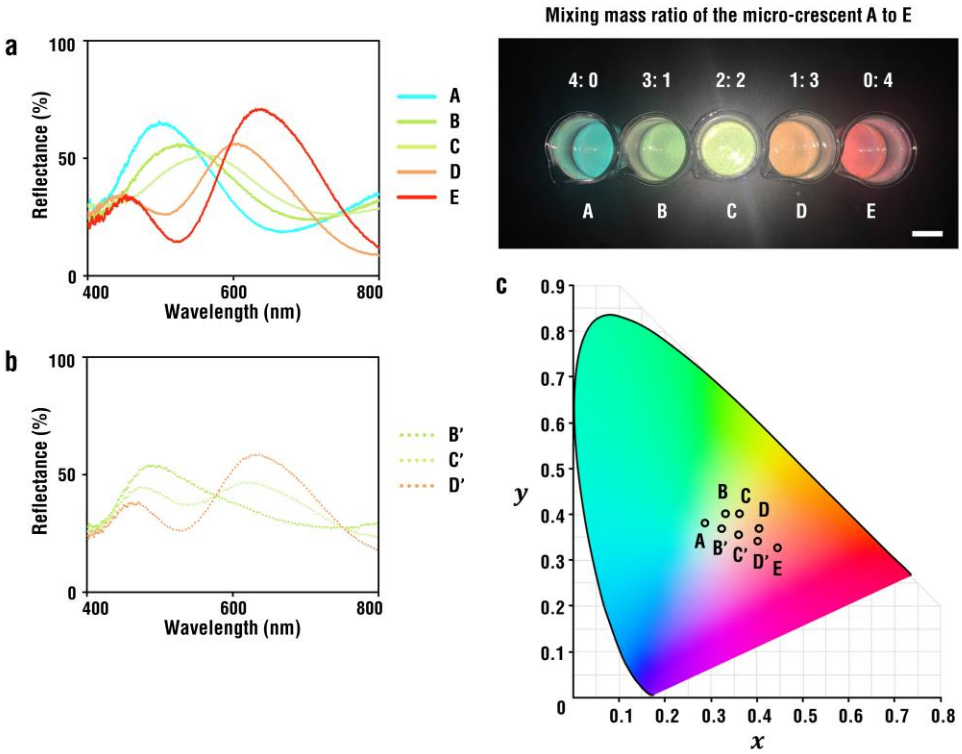

Supplementary Fig. 21 | Color mixing. (a) The reflectance spectra and photograph of the suspensions containing cyan-colored micro-crescents with a radius of 10 μm and red-colored micro-crescents with a radius of 13 μm in five different mass ratios of 4: 0 (A), 3: 1 (B), 2: 2 (C), 1: 3 (D), and 0: 4 (E). Scale bar, 20 mm. **(b)** Spectra for the mixtures with 3: 1 (B'), 2: 2 (C'), and 1: 3 (D') calculated by linear superposition of A and E. **(c)** CIE diagrams for color variation for the mixtures, where the coordinates are calculated from the experimental spectra in (a) and linear superposition in (b). The coordinates for the mixtures (B, C, and D) deviate from the line containing the points A and E, unlike the linear superposition (B', C', and D').

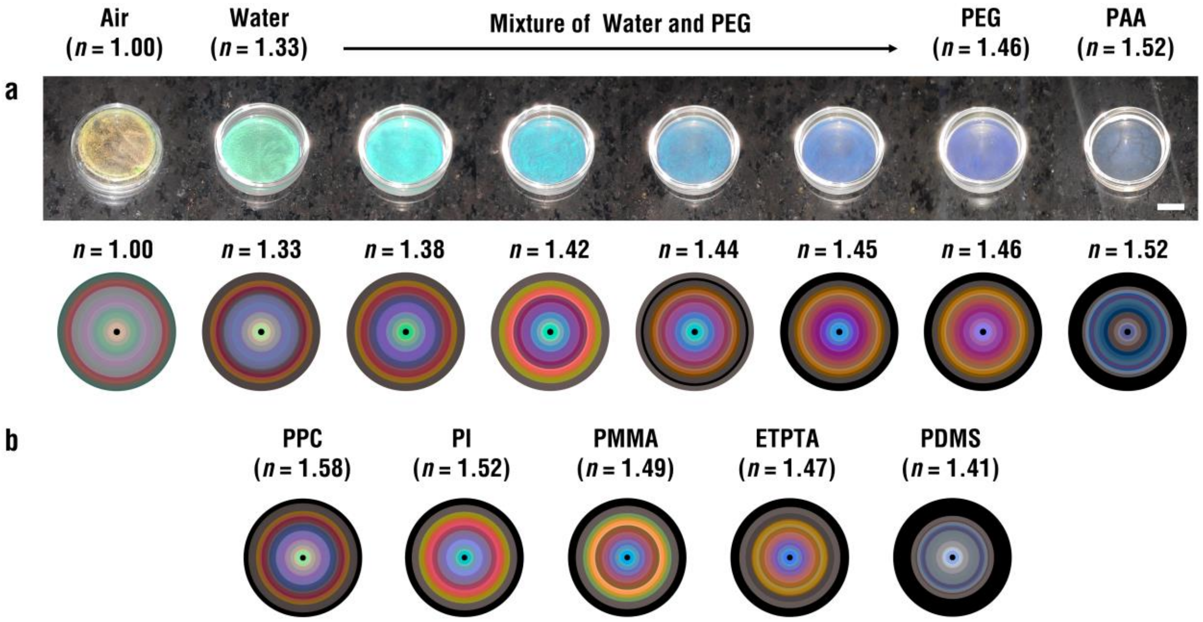

Supplementary Fig. 22 | Influence of refractive index of micro-crescent and surrounding. **(a)** Suspensions of PS micro-crescents with a radius of 18 μm in air, water, mixtures of water and PEG, and poly(acrylic acid) (PAA) and corresponding top-view color maps calculated from the model with the refractive index of the medium. Scale bar, 10 mm. As the refractive index of the medium increases, the number of available beam paths decreases because the critical angle for TIR increases, which results in the brightness reduction and color change. **(b)** Top-view color maps for the micro-crescents with five different refractive indexes in water, calculated from the model, where common hydrophobic polymers, including poly(propylene carbonate) (PPC), polyisoprene (PI), poly(methyl methacrylate) (PMMA), trimethylolpropane ethoxylate triacrylate (ETPTA), and poly(dimethyl siloxane) (PDMS), are considered as micro-crescent materials. The hue and brightness change according to the refractive index for the same shape and dimension.

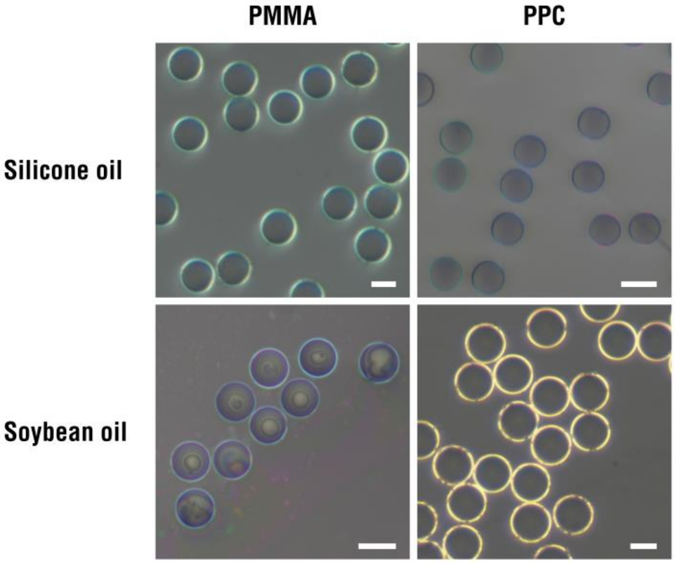

Supplementary Fig. 23 | Various sets of polymers and oils. Structurally-colored microparticles are producible from various polymers such as PMMA (n = 1.49) and PPC (n = 1.58) and various sacrificial oils, such as silicone oil and soybean oil as long as the polymers and oils have negative spreading parameters to form crescent- or lens-shaped microparticles with a proper surfactant in a continuous water phase. Scale bar, 20 μm.
