# Supplementary material for: Nanostructure-free crescent-shaped microparticles as full-color reflective pigments
Source: Nat Commun. 2023 Feb 11;14:793. doi: 10.1038/s41467-023-36482-4 (PMC9922275; doi:10.1038/s41467-023-36482-4)
Supplement: Supplementary file 2 — Description of Additional Supplementary Information [file 41467_2023_36482_MOESM2_ESM.pdf]

Description of Additional Supplementary Files

File name: Supplementary Movie 1
Description: The invariant colors with the observation angle from 0° to 75° of the pigments at retroreflection condition.

File name: Supplementary Movie 2
Description: The phoenix pattern showing colorless under ambient light but colorful under a flashlight from a smartphone.

File name: Supplementary Movie 3
Description: Optical anisotropy of the panel with well-aligned micro-crescents.

File name: Supplementary Movie 4
Description: Medium-dependent structural color of the micro-crescent array in water and air.

File name: Supplementary Movie 5
Description: Dynamic color switching for the mixture of two distinct micro-crescents with opposite magnetic moment.
